# How chimpanzees integrate sensory information to select figs

**DOI:** 10.1098/rsfs.2016.0001

**Published:** 2016-06-06

**Authors:** Nathaniel J. Dominy, Justin D. Yeakel, Uttam Bhat, Lawrence Ramsden, Richard W. Wrangham, Peter W. Lucas

**Affiliations:** 1 Department of Anthropology, Dartmouth College, 6047 Silsby Hall, Hanover, NH 03755, USA; 2 Department of Biological Sciences, Dartmouth College, 78 College Street, Hanover, NH 03755, USA; 3 School of Natural Sciences, University of California, 5200 North Lake Road, Merced, CA 95343, USA; 4 Santa Fe Institute, 1399 Hyde Park Road, Santa Fe, NM 87501, USA; 5 Physics Department, Boston University, 590 Commonwealth Avenue, Boston, MA 02215, USA; 6 School of Biological Sciences, University of Hong Kong, Pok Fu Lam Road, Hong Kong SAR, China; 7 Department of Human Evolutionary Biology, Harvard University, 11 Divinity Avenue, Cambridge, MA 02138, USA; 8 Smithsonian Tropical Research Institute, Apartado Postal, 0843-03092, Balboa, Ancon, Panama

**Keywords:** *Pan troglodytes schweinfurthii*, *Ficus*, colour vision, manual prehension, Bayesian updating

## Abstract

Figs are keystone resources that sustain chimpanzees when preferred fruits are scarce. Many figs retain a green(ish) colour throughout development, a pattern that causes chimpanzees to evaluate edibility on the basis of achromatic accessory cues. Such behaviour is conspicuous because it entails a succession of discrete sensory assessments, including the deliberate palpation of individual figs, a task that requires advanced visuomotor control. These actions are strongly suggestive of domain-specific information processing and decision-making, and they call attention to a potential selective force on the origin of advanced manual prehension and digital dexterity during primate evolution. To explore this concept, we report on the foraging behaviours of chimpanzees and the spectral, chemical and mechanical properties of figs, with cutting tests revealing ease of fracture in the mouth. By integrating the ability of different sensory cues to predict fructose content in a Bayesian updating framework, we quantified the amount of information gained when a chimpanzee successively observes, palpates and bites the green figs of *Ficus sansibarica*. We found that the cue eliciting ingestion was not colour or size, but fig mechanics (including toughness estimates from wedge tests), which relays higher-quality information on fructose concentrations than colour vision. This result explains why chimpanzees evaluate green figs by palpation and dental incision, actions that could explain the adaptive origins of advanced manual prehension.

## 1. Introduction

Figs (syconia) are swollen, urn-shaped receptacles that function simultaneously as inflorescences and fruit [1]. They define membership in the genus *Ficus* (Moraeceae), a taxon that resides in every tropical lowland rainforest and includes *ca* 800 species [2]. An outstanding feature of *Ficus* is the spectrum of plant forms: species can be hemi-epiphytes (a group that includes strangling figs and banyans), large woody climbers or trees [3], on which fig placement can be axial, cauliflorous (figs on the trunk) or geocarpic (figs on ground-level runners). A unifying trait of all figs, however, is their edibility to humans [2] and other vertebrate consumers [4].

Globally, an astounding number of vertebrates—over 1200 species—feed on figs [4]; and because pollination requires asynchronous fruiting across the population [5], edible figs are consistently present in the environment when other fruits are scarce, providing a crucial resource to frugivorous species [4]. Yet figs represent a small proportion (less than 1%) of plant diversity in a forest habitat, which suggests a keystone function [6]. Keystone taxa are those whose impact on the community or ecosystem is large, and disproportionately large relative to abundance [7]. Terborgh [6, p. 339] put it this way: ‘subtract figs from the ecosystem and one could expect to see it collapse’.

Figs are therefore central to debates on the evolutionary ecology of non-human primates. As a general rule, apes increase their consumption of figs in proportion to the decreasing availability of preferred foods (non-fig fruits) [8–15], suggesting that figs are best viewed as a reliable ‘fallback food’ [14]. This distinction between preferred foods and fallback foods is important, for it offers a theoretical basis for interpreting the evolution of primate traits that facilitate food acquisition and assimilation. Marshall & Wrangham [16] hypothesized that preferred resources are likely to drive adaptations for proficient harvesting (detection and acquisition), whereas fallback foods are likely to drive adaptations for efficient processing (chewing and digestion). Selecting figs, however, is a non-trivial task, and it has been argued [17] that geographical variation in figs has exerted a strong selective pressure on at least one harvesting trait: the primate visual system.

The central challenge for primates concerns colour and competition. Ripe figs express a wide range of external hues (typically green, yellow, orange and red [17–20]) and attract a corresponding diversity of consumers via visual and olfactory signals [20]. Further, few species demonstrate synchronized development; every phase of fig development is often present on a given tree [5]. In consequence, any primate motivated to consume figs will face a welter of sensory stimuli, and natural selection is expected to favour those individuals who develop and retain species-specific criteria that optimize fig selection [21]. Yet the basic mechanisms of how apes extract and integrate multimodal sensory information are poorly understood. Here, we focus on wild chimpanzees and how they select green figs, a potential model system for exploring the evolution of harvesting traits such as domain-specific cognition and advanced manual prehension.

### 1.1. Green figs and how chimpanzees eat them

To human observers, many figs retain a green hue throughout development. A global survey of figs found that 59 of 221 (26.7%) species are green when ripe [17]. The functional advantages of this trait are uncertain, but the retention of chlorophyll in fruits appears to offset the high respiratory costs of producing large numbers of large fruits [22]. Mammals prefer to visit larger fruit crops [23], and green aromatic figs are widely viewed as being adapted to the sensory systems of nocturnal mammals, particularly bats [18–20]. The cognitive challenge for any diurnal primate, then, is to discern the edibility of mammal(bat)-adapted figs on the basis of achromatic accessory cues.

**Figure 1.**
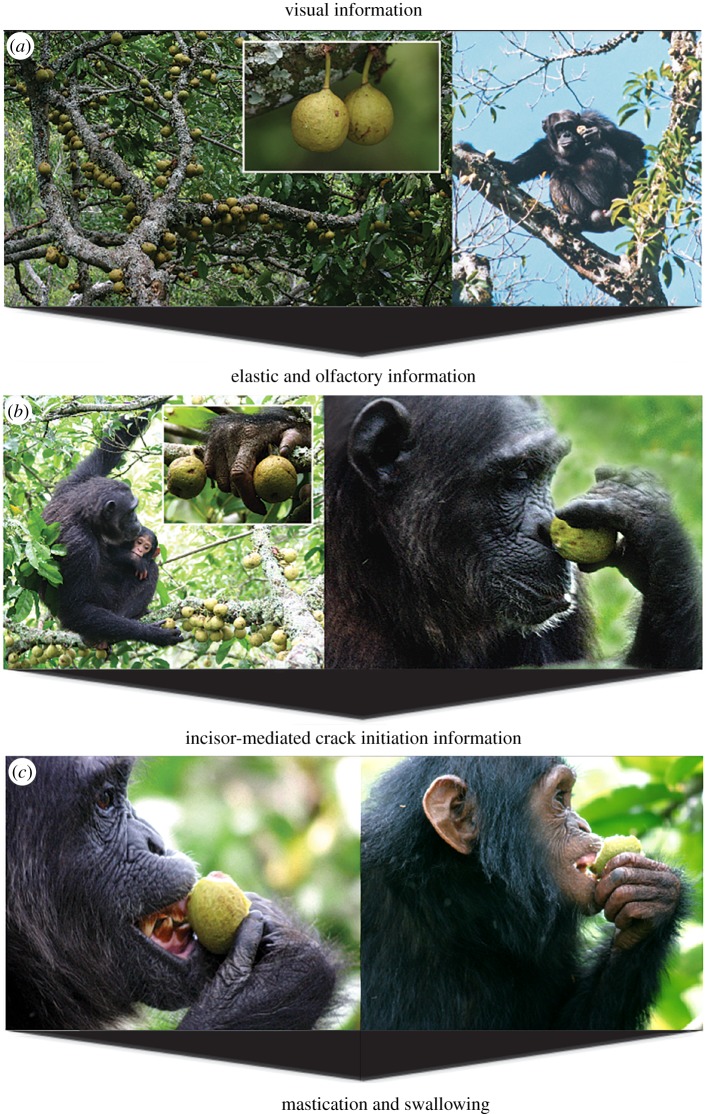
Figs of *Ficus sansibarica* and their evaluation by chimpanzees in Kibale National Park, Uganda. The mastication and swallowing of figs is preceded by successive sensory assessments: (*a*) vision, (*b*) digital palpation and/or olfaction and (*c*) incisor evaluation. Figs can be discarded at any stage of the sensory sequence (photographs by Nathaniel J. Dominy [top right only] and Alain Houle, reproduced with permission).

During the course of fieldwork in Kibale National Park, Uganda, we (N.J.D. and P.W.L.) observed chimpanzees feeding on the figs of *Ficus sansibarica* [24] (= *F. brachylepis* [25]), a large cauliflorous tree (figure 1*a*). To human observers, the golf-ball-sized figs of *F. sansibarica* are green throughout development (figure 1*a*), a pattern that frustrates efforts to estimate fig ripeness from the ground [26]. This problem of cryptic ripeness is seemingly shared with chimpanzees, who ascend trees to perform successive sensory assessments of individual figs. The deliberate and methodical nature of the behaviour is conspicuous to human observers in part because it is so familiar (see electronic supplementary material, videos S1 and S2). Sugiyama [27] observed similar manipulations (described as complicated and careful) with respect to the greenish figs of *F. mucuso* (for BBC footage, see electronic supplementary material, video S3).

Such behaviours are suggestive of information processing and decision-making [28], and they motivated the opportunistic collection and analysis of figs, with a systematic focus on *F. sansibarica*. To estimate the predictive power of different sensory modalities for estimating the fructose concentrations of figs, we measured the following attributes in the field: colour and size (to estimate visual information; figure 1*a*), Young's modulus (to estimate haptic information from manual palpations; figure 1*b*) and the crack initiation criterion, *K_IC_* (to estimate haptic information from incisal evaluations; figure 1*c*). Chimpanzees also smelled individual figs (figure 1*b*), but we were unequipped to capture olfactory volatiles. Lastly, we extracted fig contents to estimate levels of chemical deterrents (tannins) and potential nutritional rewards such as sugar and calcium concentrations [29].

Diverse animals appear capable of Bayesian updating during foraging [30], and humans behave in a manner that is consistent with Bayesian processing when engaged in visual and sensorimotor learning tasks [31]. Accordingly, we developed a Bayesian updating framework to assess information gain as chimpanzees successively view, palpate and bite the figs of *F. sansibarica*. A combination of information from multiple sensory modalities is predicted to reduce the error associated with estimating fig quality, as measured by fructose concentration, a sweet indicator of calorie content.

## 2. Methods

### 2.1. Study species and field site

We observed the foraging behaviours of chimpanzees (*Pan troglodytes*), black-and-white colobus monkeys (*Colobus guereza*), red colobus monkeys (*Procolobus badius*) and red-tailed monkeys (*Cercopithecus ascanius*) in the Kanyawara sector of Kibale National Park, Uganda (0°13′ N–0°41′ N; 30°19′ E–30°32′ E). The habitat is classified as a mix of montane moist forest and lowland rainforest with a mean annual rainfall of *ca* 1700 mm (years: 1984–1996 [32]). We employed focal animal techniques and multiple observers to maximize data collection. We switched focal animals every 10 min and collected a cumulative total of 1178 h of observational data between January and November 1999 [33–36].

### 2.2. Fig collection and measurements

Each primate species consumed figs during the study period. We observed and recorded the non-selection, rejection and ingestion of individual figs, and then we collected specimens in the following categories: (a) avoided; (b) palpated and rejected; (c) palpated, bitten (incised) and rejected; and (d) edible (defined as a fragment representing less than 50% of the ingested fruit). We collected avoided figs (category a) *in situ* by ascending trees (methods in Dominy & Duncan [37]). We collected rejected figs (categories b and c) from the ground. Edible figs (category d) were also collected from the ground, but depended on chimpanzees dropping fragments during active chewing (see electronic supplementary material, video S1). All specimens were kept in plastic polyethylene bags for conveyance to our field station, where they were refrigerated at 4°C until mechanical testing and chemical extraction at ambient temperatures.

We measured fig dimensions (length, width, thickness) when material was sufficient and used 5 mm^2^ segments of the outer surface to measure reflectance spectra [38]. We estimated the quantum catch (*Q*) of primate S-, M- and L-cone classes by multiplying each reflectance spectrum with an open-sky illuminant spectrum, and multiplying the product (the radiant spectrum) against the absorption spectra of each cone class, integrated over wavelength [33–36]. Chromaticity coordinates analogous to MacLeod–Boynton coordinates can be graphed by plotting a *y*-value of *Q*S/(*Q*L + *Q*M), which defines yellow–blueness (yellow low, blue high), against an *x*-value of *Q*L/(*Q*L + *Q*M), which defines green–redness (green low, red high) [33–36]. Such coordinates correspond with the physiological subsystems of primate colour vision, the S-cone-mediated yellow–blue subsystem (subserved by small bistratified ganglion cells) and the recently derived green–red subsystem (subserved by midget ganglion cells).

**Figure 2.**
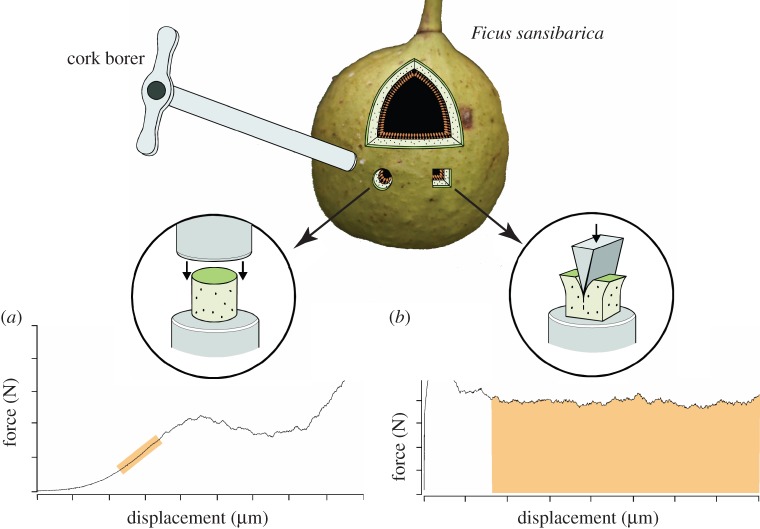
Estimates of fig mechanical properties included measures of (*a*) Young's modulus and (*b*) fracture toughness.

We used a portable mechanical tester to measure mechanical properties [38]. Samples of fig wall (mesocarp) were cut orthogonal to the outer surface and shaped with a 4 mm cork borer into right cylinders, *ca* 5 mm high. We then obtained the Young's modulus from tests on short cylinders in compression (figure 2*a*). We measured fracture toughness, i.e. the energy required for crack propagation per unit area [38], with a 15°-included angle wedge driven into small rectangular specimens cut from the fig wall (figure 2*b*). Excess work done against friction was subtracted by running the wedge through an identical displacement against the already-fractured faces of the fig tissue. After the forces during the second pass were deducted, we obtained toughness values by dividing the area under the force–deformation curve during crack growth at a force plateau (shaded in figure 2*b*) by the product of crack depth (effectively the wedge displacement) and specimen width. To account for some of the anisotropic variation within figs, mechanical measures were taken from each hemisphere and averaged. We calculated the energetic equivalent of the critical stress intensity factor (*K_IC_*) as
2.1
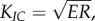

where *E* is Young's modulus and *R* is fracture toughness [39]. We view *K_IC_* here as the criterion for crack initiation and the best measure of mechanical resistance to incisal biting by chimpanzees [39].

We estimated the moisture content of figs by weighing a slice of fig wall and pressing it between two sheets of blotting paper (mass: 0.3 kg m^−2^). The dry tissue was then weighed and the percentage of expressible moisture in the fig wall calculated as the weight of absorbed moisture divided by the dry weight, multiplied by 100.

We extracted 0.1–0.5 g of fig wall in 1 : 1 deionized water : methanol and stored extracts at 4°C. Field chemical assays included a colorimetric evaluation of total phenolics and the radial diffusion assay for tannins [38]. In the laboratory, we measured molar concentrations of soluble carbohydrates with HPLC [40] and calcium concentrations with a Ca^2+^ ion selective electrode (Thermo Scientific Orion, Beverly, MA). All data from the preceding protocols were deposited in the Dryad Digital Repository (http://dx.doi.org/10.5061/dryad.m84t0).

### 2.3. Data transformations and the role of fructose

Most data were log-transformed, not only as an attempt at normalization, but also because the psychophysical response to sensory stimuli is generally linearized by this procedure [41]. We detected fructose and glucose in all figs, and low concentrations of sucrose in the figs of *F. exasperata* only. Accordingly, we focused our analyses on fructose, the predominant sugar in each sample. As fructose is also far sweeter than glucose to primates [42], we used it as an index of fig quality to primates motivated by the sense of sweetness. A practical advantage of this approach is that it allows us to use the behavioural taste thresholds of chimpanzees (40–50 mM [43,44]) to approximate the onset of fig edibility, or ripeness.

### 2.4. Bayesian model

**Figure 3.**
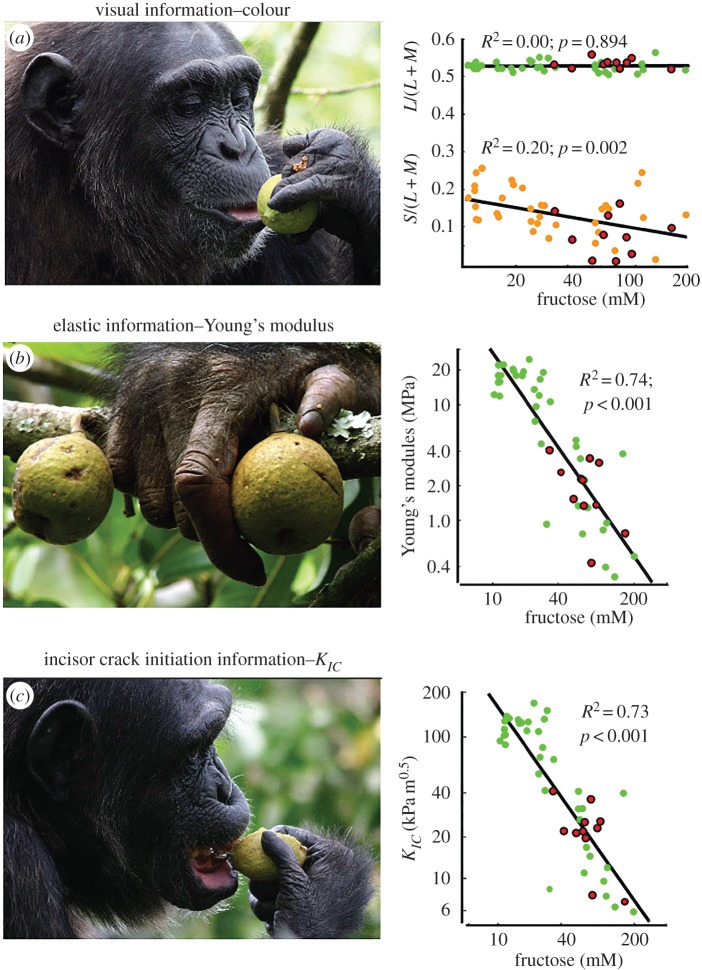
Sensory assessments and corresponding information plotted as increasing functions of fructose concentration. (*a*) fig colour on the basis green–redness [*L*/(*L* + *M*); green low, red high] and yellow–blueness [*S*/(*L* + *M*); yellow low, blue high], the two physiological subsystems of primate colour vision. (*b*) The elastic deformation of a fig is determined by its Young's modulus. (*c*) The energetic equivalent of the critical stress intensity, *K_IC_*, relates to incisal evaluation and the ease of fracture. Enclosed circles (in red) signify consumed figs (photographs by Alain Houle, reproduced with permission).

To explore how chimpanzees use and integrate sensory information to estimate the edibility of figs, we focused on the sequence of sensory assessments in figure 1 and the corresponding variables that predict fructose concentration: (i) colour (yellow–blue values), (ii) Young's modulus and (iii) *K_IC_* (see results below; figure 3). We assume that chimpanzees use information from each sensory modality to update their estimate of fructose content.

We set *Z* = *z* to be a stochastic variable describing fructose concentration (here and henceforth, uppercase notation is used to describe stochastic variables, and lowercase notation is used to describe specific values of stochastic variables), which we assume is distributed normally with an initial mean *μ*_0_ and variance *σ*_0_, such that
2.2
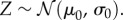



Because we want to assume initially that we know little about the distribution of fructose, we will assume that *σ*_0_ is quite large.

A foraging chimpanzee uses the different sensory modalities to obtain additional information regarding the fructose concentrations of its potential foods. Here, we establish a Bayesian framework by which knowledge of the mean fructose concentration of a potential food is updated sequentially with different kinds of sensory input, each of which relays information on fructose concentrations with different degrees of accuracy. If we consider the stochastic variable *X* = *x* that describes some form of sensory data obtained by the chimpanzee (which we also assume is normally distributed), the relationship between such data and fructose is determined by the conditional expectation and variance of fructose given the sensory data. The posterior probability distribution describing the mean fructose concentration of the food items after *n* independent sensory measurements is thus
2.3
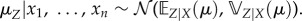



The variability of the posterior distribution is calculated as
2.4
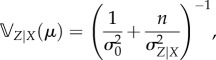

where 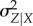 is the fructose variability conditioned on the variability of the measured sensory data, which we will describe in depth below. See reference [45] for a detailed derivation.

Chimpanzees accumulate information about fig fructose concentrations using sequential, and independent, sensory modalities. Updating the frequency distribution that describes the mean fructose concentration from *m* independent observations *y*_1_, … , *y_m_* using a second sensory mode modifies the variance of the posterior probability distribution describing fructose concentration such that
2.5
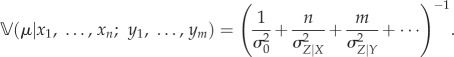

One observes from equation (2.5) that additional data always serves to lower 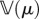, though determining the magnitude of this decrease requires knowledge of the conditional variability of fructose concentrations with the respective sensory data gathered for each sensory mode. Thus, understanding the relationships between different types of sensory data with fig fructose concentrations will enable determination of 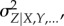 and this will allow us to quantify how the uncertainty of fructose concentration is lowered as a foraging animal uses different senses to identify the quality of potential foods.

Field data show that yellow–blue frequencies of figs are linearly related to fructose concentrations with the slope *a*, an offset *b*, and a Gaussian noise term 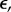 multiplied by the amplitude of noise *σ_X_*_|*Z*_ that describes the variability of the yellow–blue frequency data given the variability in fructose. The relationship between sensory data and the fructose concentration of figs is thus 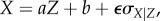 such that the expectation and variability are
2.6
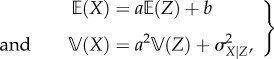

where 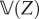 is the initial (prior) uncertainty of fructose concentration, which we assume is large and write henceforth as 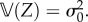 First, we define the correlation between data gathered from a given sensory mode *X* and fructose concentrations *Z* as *ρ_X_*, and this determines the ability of a set of sensory data to provide information on the nutritional quality of food. Second, the inherent variability of sensory data given variability of fructose concentrations, *σ_X_*_|*Z*_ constrains the potential uncertainty in using a given sensory mode to measure fructose. Correlation between sensory data and fructose, as well as the conditional uncertainty of sensory data, are directly related as
2.7
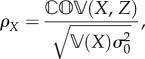

where 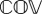 is the covariance. Plugging in the relationships defined in equation (2.6), we can simplify this to
2.8
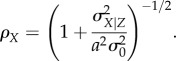



Because the slope between sensory data and fructose 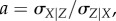 we can rewrite equation (2.8) to define the conditional variability of fructose given sensory data in terms of the correlation between the two, as well as the prior variability of fructose, such that
2.9
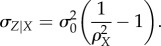



Finally, we can rewrite the posterior variance of fructose concentrations in terms of the correlations between fructose and different sources of sensory data, such that
2.10
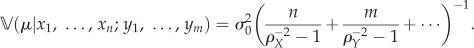

where the (…) designates future updates to the posterior variability using different sources of sensory data from alternative modalities.

We are interested in the amount of information that is acquired by sequential sensory data as a chimpanzee evaluates its potential food with different sensory modalities. To determine information gain, we must first calculate the differential entropy 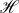 of the posterior normal distribution after the chimpanzee obtains some set of sensory data, where 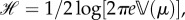 where *e* is Euler's number [46]. Information is the difference in uncertainty after measurements were made relative to the uncertainty before measurements were made [46]. Thus, in this context, information 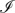 is formally calculated by measuring the change in differential entropy before gathering data *X* (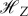) relative to the differential entropy after gathering data *X* (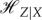), such that 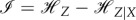 [46].

## 3. Results

We observed chimpanzees, black-and-white colobus monkeys, red colobus monkeys and red-tailed monkeys for 58, 378, 412 and 330 h, respectively, and recorded 818, 131, 127 and 174 min of fig-feeding, respectively. The species consumed were *Ficus exasperata*, *F. cyathistipula*, *F. natalensis*, *F. pilosula* and *F. sansibarica*. A majority of fig specimens (51 of 84) were of *F. sansibarica*, the species that elicited manual palpations. The palpations (with the volar pad of the thumb and lateral side of the index finger; figure 1*b*) were rapid, taking a mean (±1 s.d.) of 1.43 ± 0.34 s from initial arm movement to fig release (*n* = 25 filmed events). This assessment of Young's modulus was about four times faster than the average time required to assess *K_IC_*, i.e. to detach, and then bite, a fig before discarding it (5.83 ± 1.24 s; *n* = 13 filmed events). No clear video records were obtained for monkeys, but the colobines lack thumbs and evaluated figs directly with the mouth.

Another feature of colobus monkeys is their large sacculated stomach, a trait related to a diet of leaves and unripe fruits. Figs consumed by the colobine monkeys (*n* = 13) differed from those consumed by chimpanzees and red-tailed monkeys (*n* = 19), with higher Young's modulus and *K_IC_* values (*p* = 0.01 or better) and much lower fructose concentrations (*p* < 0.001). The tannin contents were also higher, though not significantly. Together, these findings support the view that colobine monkeys target fruits with different sensory attributes (see electronic supplementary material, figure S1*a*).

Figure 3 illustrates the developmental sequence of *F. sansibarica*. We detected no variation in green–redness as an increasing function of fructose concentration, but we did detect a significant increase in yellowness (figure 3*a*). Yellowness, however, did not distinguish between figs that were rejected or consumed (figure 3*a*), highlighting the noise of this cue and the need for supplemental information. Relative size was a potential visual cue, but we could not estimate the sizes of consumed figs on the basis of dropped fragments; however, the mean diameter of avoided figs (39.6 ± 12.8 mm) did not vary with fructose concentration (*p* > 0.05), suggesting that fig size was an unreliable visual cue. The Young's modulus of the fig wall varied significantly as a negative function of fructose concentration (figure 3*b*). A similar relationship was observed with *K_IC_* (figure 3*c*), a variable that relates to the ease of incisor-mediated tissue fracture. Lower values of *K_IC_* are necessary to release moisture, which, in turn, is necessary to deliver soluble sugars to taste receptors. We found that the moisture content of figs varied significantly as a positive function of fructose concentration (see electronic supplementary material, figure S1*b*)

We analysed a subset of figs—those that chimpanzees discarded after incisal biting (*n* = 9) versus those that they consumed (*n* = 11)—and found that consumed figs had significantly lower tannin levels (*p* < 0.05). We detected no evidence of calcium variation during development or selection by chimpanzees. The mean Ca^2+^ concentration of consumed figs (3.5 ± 5.6 mM) was marginally lower than that of rejected figs (4.9 ± 5.6 mM), but the difference did not reach statistical significance.

We evaluated the information gained when chimpanzees used the successive sensory modalities shown in figure 1. We compared the information gained from the known sequence of sensory modalities to a baseline sequence where it is assumed that all sequential data come from vision such that they all have correlations equivalent to that of yellow–blue frequencies and fructose. The difference between the information gained from the observed sensory modalities compared with the baseline thus reveals the information benefits of palpating/biting fruits versus a reliance on visual cues alone.

**Figure 4.**
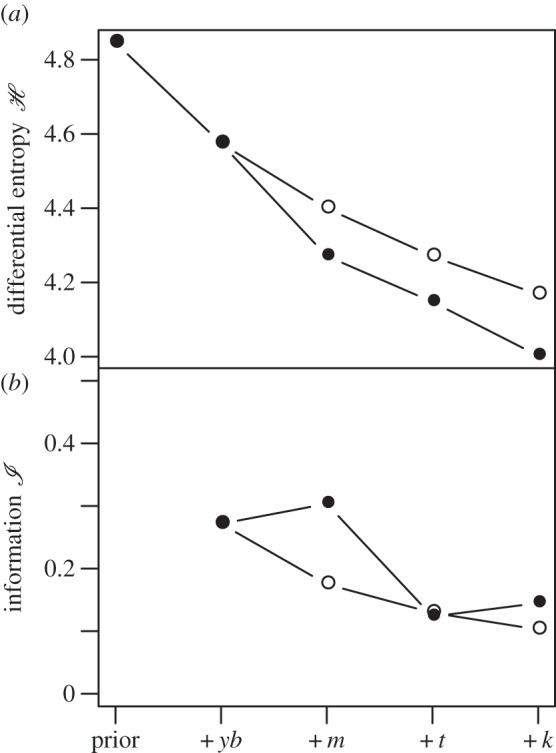
(*a*) Measures of the differential entropy (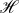) and (*b*) information gain (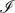) for the probability distribution describing the known fructose concentrations of figs (the following are additive) before observations (the prior), combined with observations of yellow–blue frequencies (+*yb*), combined with observations of Young's modulus (+*m*), combined with observations of toughness (+*t*), combined with observations of *K*_*IC*_. Filled circles show differential entropy and information gained from the known sequence of sensory modalities, and open circles represent a baseline sequence where it is assumed that all data are visual and have correlations equivalent to that of yellow–blue frequencies and fructose.

The results of our analysis show that—for both the baseline and the actual sequence of sensory modalities—successive evaluation of fig properties always serves to decrease the differential entropy of the posterior distributions describing the mean fructose concentration of observed figs. This means that the variance of this distribution is similarly lowered with successive measurement such that the observer is gaining information by decreasing uncertainty (figure 4*a*). Quantified in terms of information 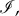 we observe that chimpanzees gain more information by both touching (informing elasticity) and biting (informing hardness; figure 4*b*). Toughness is also evaluated during handling; however, the information gained is similar to that gained by vision alone.

## 4. Discussion

The present analysis is focused primarily on chimpanzees and the green figs of *F. sansibarica*. We observed successive sensory behaviours and found that integrated sensory inputs—from visual inspection to palpation to incisal evaluation—are more informative than visual cues alone. Our primary conclusions are threefold: (i) chimpanzees demonstrate domain-specific cognitive behaviours when foraging on green figs; (ii) these modular behaviours are well suited to collecting information related to fig quality, albeit with different levels of certainty and (iii) the integration of successive sensory inputs can reduce uncertainty and therefore maximize information concerning the caloric value of figs. Value, however, is a subjective concept that depends in part on the digestive physiology and energetic demands of the consumer.

Chimpanzees are said to have high-quality diets compared with monkeys [47]. This distinction appears unrelated to fruit-species composition, but rather the systematic selection of individual fruits with higher levels of soluble carbohydrates and lower levels of fibre [47]. Such a finding agrees well with our limited data comparing fructose and toughness, but the pattern is difficult to understand given that high-quality fruits should hold equal attraction for chimpanzees and cercopithecine monkeys. It is tempting to suggest (on the basis of figure 4*b*) that advanced manual prehension gives chimpanzees a decisive advantage when harvesting greenish figs, such as those of *F. sansibarica*. Recall that figs are crucial fallback foods that sustain chimpanzees and other apes at times when preferred foods are scarce [14]. Figs, then, may have exerted a disproportionately strong selective pressure on chimpanzees, particularly their high level of manual [48] and somatosensory intelligence [49].

The precision grip of humans is unparalleled among vertebrates, a fact that is often linked to the adaptive advantages of complex tool use [50–54]. Perhaps surprisingly, much less attention has been focused on the mechanosensory adaptations that preceded this level of manual prehension and dexterity [55]. Several plant foods in the diets of gorillas and chimpanzees are known to command complex manipulations during harvesting [56–58]; however, there is little evidence of modular sensory evaluations or thoughtful deliberation. The present findings are germane to this issue as they demonstrate the nutritional advantages of assessing elastic deformation by palpation, an underappreciated food-handling task that requires advanced visuomotor control. It also saves time—palpating figs was about four times faster than assessing *K_IC_*—suggesting that chimpanzees enjoy a substantial foraging advantage over competitors that rely solely on visual and oral information, such as birds and monkeys.

The behaviour of chimpanzees towards green figs bears a stronger resemblance to cryptic prey detection than it does a mutualism between plant and seed disperser, suggesting that memory (or search image, as Tinbergen put it [21]) contributes to palpation as much as the requisite morphology and neuroanatomy. A crucial point is that our analysis naturally simulates learning by using a Bayesian updating approach. This framework helps explain why an individual chimpanzee might integrate two comparable sources of mechanical information—for example, palpation in tandem with incisal evaluation. The added value of reduced uncertainty is expected to vary according to the internal state of the individual, e.g. reproductive status, health condition or level of satiety.

Ultimately, it is desirable to explore the fitness consequences of different food-handling behaviours. The trade-offs between information-processing and food intake rate are naturally stochastic, and a foraging individual must weigh choices based on uncertain information. It follows that any anatomical, behavioural or cognitive trait that minimizes uncertainty will confer a selective advantage, and it is tempting to view the hands of chimpanzees as mechanical testing instruments. The advantage of this outlook is that it offers a fresh perspective on the evolution of skilled forelimb movements. Tool use is perhaps best viewed as the exaptation of a hand that was itself a tool for evaluating cryptic foods.

## Supplementary Material

Figure S1

## Data accessibility

All data were deposited in the Dryad Digital Repository (http://dx.doi.org/10.5061/dryad.m84t0).
